# Long-Term Therapeutic Plasma Exchange to Prevent End-Stage Kidney Disease in Adult Severe Resistant Henoch-Schonlein Purpura Nephritis

**DOI:** 10.1155/2015/269895

**Published:** 2015-11-03

**Authors:** Patrick Hamilton, Olumide Ogundare, Ammar Raza, Arvind Ponnusamy, Julie Gorton, Hana Alachkar, Jamil Choudhury, Jonathan Barratt, Philip A. Kalra

**Affiliations:** ^1^Renal Department, Salford Royal NHS Foundation Trust, Salford, Greater Manchester M6 8HD, UK; ^2^Histopathology Department, Salford NHS Foundation Trust, Salford, Greater Manchester M6 8HD, UK; ^3^John Walls Renal Unit, Leicester General Hospital, Gwendolen Road, Leicester LE5 4PW, UK

## Abstract

A 27-year-old man presented with a palpable purpuric skin rash and joint and abdominal pain in April 2010. He had acute kidney injury and his creatinine quickly deteriorated to 687 *μ*mol/L, with associated nephrotic range proteinuria. Kidney biopsy showed crescentic Henoch-Schonlein nephritis. He was treated with intravenous cyclophosphamide and prednisolone despite which his renal function deteriorated; he required haemodialysis for a short duration and seven sessions of therapeutic plasma exchange (TPE). Renal function improved, but after discharge from hospital he suffered 2 further relapses, each with AKI, in 4 months. Cyclophosphamide was not effective and therefore Rituximab was introduced. He initially had a partial response but his renal function deteriorated despite continued therapy. TPE was the only treatment that prevented rapid renal functional deterioration. A novel long-term treatment strategy involving regular TPE every one to two weeks was initiated. This helped to slow his progression to end-stage kidney disease over a 3-year period and to prolong the need for renal replacement therapy over this time.

## 1. Background

Henoch-Schonlein Purpura (HSP) is a nonthrombocytopenic, purpuric, and systemic vasculitis characterised by the deposition of immune complexes containing IgA in small venules, capillaries, and arterioles. It classically presents with the tetrad of skin, joint, and gastrointestinal and renal manifestations with approximately 90% of patients under the age of 10 years [1, 2]. In adults, although rare, it represents a more severe clinical syndrome, with a higher frequency of renal involvement [3–5]. Various treatment modalities have been used, including steroids and immunosuppression, but there is currently no consensus on the most effective treatment [6]. Therapeutic plasma exchange (TPE) has also been used in adults and children for a number of years but has been limited to short-term therapy only [7–22]. Here we present a young adult with HSP and rapidly progressive kidney disease in whom we established long-term regular TPE for over three years to successfully hold off progression to end-stage renal disease (ESRD).

## 2. Case Report

**Figure 1:**
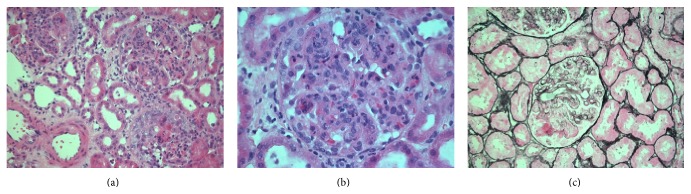
First native renal biopsy. (a and b) H&E slides from showing diffuse proliferative changes in all glomeruli, with neutrophils and foci of fibrinoid necrosis associated with epithelial crescents. (c) Silver stain showing obliteration of capillary loops, fibrinoid necrosis, and double contouring. Immunofluorescence showed a prominent granular positivity within the mesangium and to some extent within the membranes for IgA and C3. Oxford classification *M* = 1, *E* = 1, *S* = 0, and *T* = 0.

In April 2010 a 27-year-old male with well controlled asthma presented to his local hospital with abdominal pain, palpable purpuric skin rash, bloating, sore throat, and joint swelling. He was diagnosed with HSP and commenced on oral steroids. Creatinine on admission was 104 *μ*mol/L but three weeks later he transferred to our tertiary renal centre with a creatinine of 181 *μ*mol/L, serum albumin 19 g/L, and haematoproteinuria, with a urinary protein: creatinine ratio (uPCR) of 6.19 g/g (700 g/mol). A renal immunological screen and ultrasound of the renal tract (including renal vein Doppler sonography) were normal. IgA levels were normal (2.21 g/L) with no evidence of IgA paraprotein. He had three pulses of methylprednisolone, and an urgent renal biopsy revealed appearances suggestive of HSP/IgAN with a prominent membranoproliferative pattern (Figure 1). Three of 15 (20%) glomeruli showed foci of fibrinoid necrosis associated with epithelial crescents and no evidence of fibrosis. Immunofluorescence demonstrated prominent granular positivity within the mesangium and within the membranes for IgA and C3. Electron microscopy showed prominent mesangial, paramesangial, and subendothelial deposits with associated patchy effacement of epithelial foot processes. The basement membranes appeared unremarkable. Oxford classification was *M* = 1, *E* = 1, *S* = 0, and *T* = 0. A mesangial hypercellularity score of 1 was originally shown to be an independent risk factor of renal decline [23]. In the Oxford classification validation study, the endocapillary hypercellularity score was shown to be associated with worsening renal function [24]. In the original Oxford classification study and the validation study both segmental glomerulosclerosis (*S*) and tubular atrophy/interstitial fibrosis (*T*) score were also shown to be associated with a poor renal outcome although these were not present on our patient's initial biopsy.

With the severity of the biopsy features and deteriorating renal function he was escalated to intravenous (IV) cyclophosphamide which stabilised his creatinine at around 280 *μ*mol/L for one week before deteriorating rapidly to 687 *μ*mol/L, after which he started haemodialysis and TPE. His creatinine improved to 300 *μ*mol/L after 7 sessions of TPE; he stopped haemodialysis and was discharged on oral steroids, Ramipril, and IV cyclophosphamide. The IV cyclophosphamide was given monthly for 6 months, starting at a dose of 1200 mg (0.75 g/m^2^), with a reduction to 1000 mg following the initial infusion due to renal function decline.

In July and August 2010 he was admitted twice with relapses characterised by acute kidney injury (AKI, creatinine 519 *μ*mol/L); each of these relapses was heralded by a worsening of his rash but with no reduction in serum albumin or deterioration in uPCR (serum albumin 37 g/L and uPCR 2.40 g/g (271 g/mol) in August). During each episode of AKI, other causes such as infection, nephrotoxic therapy, or change in therapy were ruled out and hence the episodes were attributed to active HSP disease; further evidence for this was provided by the fact that the renal function improved following TPE.

A repeat biopsy during the first relapse showed less severe acute glomerular lesions compared to his first biopsy: 3/30 sclerosed glomeruli but predominantly global mesangial hypercellularity, 13% (4/30) crescent formation, and mild diffuse interstitial fibrosis with some tubular loss. Immunofluorescence showed predominantly mesangial IgA deposits with weak IgG and C3 staining.

**Figure 2:**
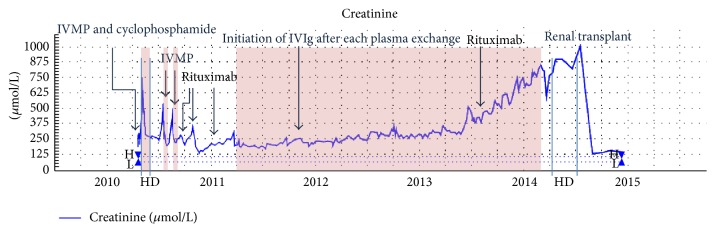
Therapy timeline with serum creatinine level from presentation to December 2014. Shaded area represents TPE therapy. IVMP, intravenous methylprednisolone; HD, haemodialysis; IVIg, intravenous immunoglobulin; PLEX, TPE.

He received IV methylprednisolone followed by oral prednisolone, continuation of IV cyclophosphamide (6 pulses by 14 weeks), and 7 sessions of TPE, again with an improvement in his creatinine. He relapsed two weeks later with worsening leg rash, requiring IV methylprednisolone, two sessions of haemodialysis, and 4 sessions of TPE. Following this he had Rituximab which led to an improvement both clinically and biochemically with creatinine dropping to 181 *μ*mol/L and uPCR to 0.88 g/g (100 g/mol) (Figure 2). His immunoglobulin levels were normal but dropped after commencing Rituximab with IgA levels falling to 0.54 g/L. From July 2010 to January 2011 he had a total of six doses but four weeks after his last dose his renal function was clearly deteriorating again. He initially received 1 g Rituximab in July 2010 followed by three doses of 700 mg at weekly intervals with the last dose on 29 September 2010. He received a further 700 mg Rituximab in October 2010 and another 1 g in January 2011.

Given the previous beneficial responses to TPE it was felt that regular long-term TPE was the most likely way to safely avoid further episodes of AKI. No evidence base was available to guide frequency and so he was empirically treated with a session every 1 or 2 weeks depending on symptoms and response of creatinine. The regular TPE regime was commenced 11 months after initial presentation and he was maintained on 5 mg prednisolone and Ramipril daily which kept his blood pressure well controlled.

In view of the long-term treatment plan he had an arteriovenous fistula fashioned to facilitate the TPE. Joint care with the immunology team helped to reduce the infection risk and he received daily prophylactic azithromycin, as well as intravenous immunoglobulin (initially 15 g but increased to 20 g due to low serum IgG levels in January 2012) with every session.

For 3 years, between March 2011 and March 2014, he had a total of 108 sessions of TPE with no further AKI episodes. There was however a steady decline in renal function (approximately 9 mL/min/1.73 m^2^/year) and persistent haematoproteinuria with proteinuria in the range of 1.77–2.65 g/g (200–300 g/mol) with no significant improvement if the frequency of TPE was increased. Over this period his main extrarenal manifestation of leg rash settled.

From the middle of 2013 there was acceleration in his renal decline and associated rise in uPCR to 6.44 g/g (729 g/mol) but there were no extrarenal manifestations (Figure 2). This could not be halted despite increasing his TPE frequency to alternate days and reintroduction of Rituximab in July and October 2013 (1 g for each infusion). He started regular haemodialysis in March 2014 and had a live related renal transplant from his brother in August 2014. The graft functioned well from the outset with creatinine stabilizing at 150 *μ*mol/L.

## 3. Discussion

Less than 10% of cases of HSP occur in adults, but this condition can have catastrophic implications with up to 11% reaching ESRD and 13% exhibiting severe renal impairment (creatinine clearance < 30 mL/min) [1–3]. Most studies investigating HSP in adults have been limited by small numbers but the consistency of disease severity and the nature of renal involvement is striking [3–5].

Unfortunately treatment options are limited, with little convincing evidence for immunosuppressive therapy; other than steroids, the Kidney Disease: Improving Global Outcomes (KDIGO) guidelines do not suggest the addition of immunosuppressive agents [6] and a meta-analysis concluded that data for any interventions that might improve kidney outcomes were sparse except for short-term prednisolone [25]. Rituximab has shown some promise although at present there have only been three case reports in adult onset HSP, albeit all successful in controlling AKI and extrarenal manifestations and preventing ESRD [26–28].

The use of TPE alone or in combination with immunomodulation therapies in the acute phase of HSP is well reported in the literature [7–22]. However to the best of our knowledge there has been no previous report of the long-term use of TPE to prevent the onset of ESRD. Our patient received over 100 sessions of TPE in the more chronic progressive phase of his condition.

Since Berger and Hinglais described the mesangial deposition of IgA, the importance of this immunoglobulin in the disease pathogenesis has become ever clearer [29]. The body produces IgA at a daily rate which is more than all other immunoglobulins combined, but due to its short half-life and loss in secretions it is the second most prevalent class of antibody after IgG [30]. In patients with HSP and IgAN there appears to be abnormal glycosylation of polymeric IgA1 [31–33] with these molecules having a predilection for self-aggregation and for combining with IgG molecules to form antigenic circulating IgA-containing complexes [34, 35]. These circulating complexes, and in particular the high molecular weight complexes and those with high levels of aberrantly glycosylated IgA1, become deposited in the glomerular mesangium with high affinity and with subsequent stimulation of cellular proliferation, cytokine release, immune cell infiltration, and glomerular injury [36–40].

Following the discovery of the presence of circulating immune complexes in HSP over 30 years ago [36], reports began to emerge of the use of TPE in treatment of HSP. The benefit of this therapy lies in its ability to remove circulating immune complexes as found in HSP and IgAN [8]. Since that time, TPE has been used successfully in both adults and children and also for conditions associated with HSP such as cerebral vasculitis, intracerebral haemorrhage, haemorrhagic pancolitis, and skin manifestations but generally as a temporary measure [12, 13, 16, 17, 19, 21, 41]. The successful outcomes described in the literature may be due to the high proportion of patients, children especially, whose disease is self-limiting and also because of a degree of positive reporting bias. Given the 6-day half-life of IgA molecules, it could be hypothesised that in patients with a more severe phenotype of the disease more regular and long-term therapy with TPE should be necessary to remove the circulating immune complexes. Here we have shown that, in a patient with rapidly progressive renal disease resistant to traditional therapy, the long-term use of TPE can hold off the need for dialysis for a number of years.
